# Gastric Cancer Cell-Derived Exosomal GRP78 Enhances Angiogenesis upon Stimulation of Vascular Endothelial Cells

**DOI:** 10.3390/cimb44120419

**Published:** 2022-12-06

**Authors:** Kanako Iha, Akane Sato, Hsin-Yi Tsai, Hikaru Sonoda, Satoshi Watabe, Teruki Yoshimura, Ming-Wei Lin, Etsuro Ito

**Affiliations:** 1 Department of Biology, Waseda University, Shinjuku, Tokyo 162-8480, Japan; 2 Department of Medical Research, E-Da Hospital/E-Da Cancer Hospital, Kaohsiung 82445, Taiwan; 3 School of Pharmacy, Kaohsiung Medical University, Kaohsiung 80708, Taiwan; 4 Hakarel Inc., Ibaraki, Osaka 567-0085, Japan; 5 Waseda Research Institute for Science and Engineering, Waseda University, Shinjuku, Tokyo 169-8555, Japan; 6 School of Pharmaceutical Sciences, Health Sciences University of Hokkaido, Tobetsu, Hokkaido 061-0293, Japan; 7 Department of Nursing, College of Medicine, I-Shou University, Kaohsiung 82445, Taiwan; 8 Regenerative Medicine and Cell Therapy Research Center, Kaohsiung Medical University, Kaohsiung 80708, Taiwan; 9 Graduate Institute of Medicine, Kaohsiung Medical University, Kaohsiung 80708, Taiwan

**Keywords:** angiogenesis, exosome, gastric cancer cell, GRP78, ultrasensitive ELISA, vascular endothelial cell

## Abstract

Exosomes containing glucose-regulated protein 78 (GRP78) are involved in cancer malignancy. GRP78 is thought to promote the tumor microenvironment, leading to angiogenesis. No direct evidence for this role has been reported, however, mainly because of difficulties in accurately measuring the GRP78 concentration in the exosomes. Recently, exosomal GRP78 concentrations were successfully measured using an ultrasensitive ELISA. In the present study, GRP78 concentrations in exosomes collected from gastric cancer AGS cells with overexpression of GRP78 (OE), knockdown of GRP78 (KD), or mock GRP78 (mock) were quantified. These three types of exosomes were then incubated with vascular endothelial cells to examine their effects on endothelial cell angiogenesis. Based on the results of a tube formation assay, GRP78-OE exosomes accelerated angiogenesis compared with GRP78-KD or GRP78-mock exosomes. To investigate the mechanisms underlying this effect, we examined the Ser473 phosphorylation state ratio of AKT, which is involved in the angiogenesis process, and found that AKT phosphorylation was increased by GRP78-OE exosome application to the endothelial cells. An MTT assay showed that GRP78-OE exosome treatment increased the proliferation rate of endothelial cells, and a wound healing assay showed that this treatment increased the migration capacity of the endothelial cells. These findings demonstrated that GRP78-containing exosomes promote the tumor microenvironment and induce angiogenesis.

## 1. Introduction

Glucose-regulated protein 78 (GRP78) is involved in various aspects of cancer, such as invasion and metastasis [1], and GRP78 inhibitors are now considered potential cancer therapy targets [2]. In the tumor microenvironment, GRP78 contained in exosomes, transport vesicles of about 100-nm in size, is released from cancer cells [3], where it affects the surrounding cells and induces cancer malignancy [4]. Recent studies suggest that GRP78 also plays an important role in angiogenesis. For example, angiogenesis is suppressed in GRP78-hetero-knockdown (KD) mice [5]. Exosomes collected from cancer cells, with or without a GRP78 inhibitor applied to vascular endothelial cells exhibit different effects on angiogenesis [6,7]. Although these studies provide indirect evidence that GRP78 promotes angiogenesis, there has been no direct evidence provided to indicate that GRP78-containing exosomes released from cancer cells act on surrounding vascular endothelial cells, thereby evoking angiogenesis. A primary reason for the lack of direct evidence is the difficulty in quantifying the trace amounts of GRP78 in the exosomes.

Our recent studies using an ultrasensitive ELISA with thionicotinamide-adenine dinucleotide (thio-NAD) cycling, however, enabled us to measure the concentration of GRP78 in exosomes derived from gastric cancer cells (AGS and MKN45) at the sub-attomolar level [3,4]. The migration and proliferation of cancer cells incubated with high-GRP78-containing exosomes were increased based on the results of both an MTT assay and a wound healing assay [4]. Furthermore, GRP78 was successfully quantified in both the lumen and membrane fractions of exosomes obtained from cancer cells [3]. Thus, we are now able to examine whether the tumor microenvironment, including angiogenesis, is differentially altered by various concentrations of GRP78 in exosomes derived from cancer cells.

In the present study, exosomes were collected from AGS cells (a gastric cancer cell line) with overexpression of GRP78 (OE) or knockdown of GRP78 (KD), and then incubated with immortalized vascular endothelial cells. The findings provided direct evidence that GRP78 in the exosomes released from cancer cells acted on vascular endothelial cells to induce angiogenesis.

## 2. Materials and Methods

### 2.1. Cell Culture

Three kinds of GRP78-transfected gastric cancer AGS cells were purchased from the National RNAi Core Facility (RNA Technology Platform and Gene Manipulation Core, Academia Sinica, Taipei, Taiwan) as follows: (1) AGS/GRP78 + (AGS with GRP78-Bip-pLAS2w; referred to hereafter as ‘GRP78-OE’ AGS); (2) AGS/shGRP78 (AGS with shRNA, shHSPA5, clone ID: TRCN218611; referred to hereafter as ‘GRP78-KD AGS’); and (3) AGS/nc (AGS with scrambled shRNA, shLacZ1339, shLacZ, clone ID: TRCN231722; referred to hereafter as ‘GRP78-mock’ AGS). Transfected AGS cells were cultured using RPMI1640 (Nacalai Tesque, Kyoto, Japan) supplemented with 10% FBS, penicillin-streptomycin mixed solution (Nacalai Tesque), and 0.75 mg/mL puromycin dihydrochloride (Cat # ant-pr-1, InvivoGen, San Diego, CA, USA). The growth rates of these 3 cell types were similar.

Immortalized human umbilical vein endothelial HUEhT-1 cells were obtained from the JCRB Cell Bank (JCRB1458). HUEhT-1 cells are HUVEC cells with forced expression of telomerase that exhibit a tube formation similar to normal HUVEC cells [8]. HUEhT-1 cells were cultured using MCDB131 (Thermo Fisher Scientific, Gibco, Waltham, MA, USA) supplemented with 10% FBS, 0.03 g/L endothelial cell growth supplement (Corning, Corning, NY, USA), 5 μg/mL heparin (Sigma-Aldrich, St. Louis, MO, USA), and 10 mM l-glutamine (Nacalai Tesque). All the cells were grown in a humidified incubator at 37 °C with 5% CO_2_.

### 2.2. Preparation and Confirmation of Exosomes

Exosomes were extracted from the gastric cancer cell culture media for AGS cells using Total Exosome Isolation Reagent (from cell culture media; Cat # 4478359, Thermo Fisher Scientific, Invitrogen, Waltham, MA, USA). The transfected AGS cells were cultured for 48 h in RPMI1640, without FBS (conditioned medium). The culture media were centrifuged at 2000× *g* for 30 min at 4 °C to remove cells and cell debris. The supernatant was filtered through a 0.22-µm filter and ultra-filtered to remove apoptotic bodies. The filtered supernatant was ultrafiltered using a 100-kDa cutoff Amicon ultra-15 centrifugal filter (UFC910024; Merck Millipore, Burlington, MA, USA). The ultrafiltration tube was centrifuged at 4000× *g* for 30 min. A Total Exosome Isolation Reagent (from cell culture media, 4478359; Invitrogen) was added to the ultrafiltered conditioned media. After overnight incubation at 4 °C, the mixture was centrifuged at 10,000× *g* at 4 °C for 1 h to collect the exosome pellets. The exosome pellets were resuspended in 100 µL of RPMI1640 without FBS.

The exosome pellets were diluted at 1:10,000 with PBS. The exosome size was examined with a nanoparticle tracking video-microscope, ZetaView (Particle Metrix, Inning am Ammersee, Germany), with ZetaView software version 8.05.14 (Particle Metrix).

### 2.3. Western Blotting

Western blotting was performed for three kinds of GRP78-transfected gastric cancer AGS cells (AGS-OE, AGS-KD, and AGS-mock) to examine the expression level of GRP78. The cells were collected and washed with PBS. The total protein samples were extracted and protein concentrations were measured using Bio-Rad Bradford Protein Assays (Bio-Rad, Hercules, CA, USA). Equal quantities of total protein were separated by electrophoresis on BOLT BISTRIS PLUS 4–12% SDS-PAGE (Thermo Fisher Scientific) and transferred onto PVDF membranes. The membranes were incubated in blocking buffer (Bio-Rad) for 30 min at room temperature, and then overnight at 4 °C with the primary antibody of the anti-GRP78 antibody (1:1000; Cat # 3177S, Cell Signaling Technology, Danvers, MA, USA) or anti-actin antibody (1:1000; Cat # MAB1501, Merck Millipore). After washing with PBS containing Tween 20 (PBS-T), the horseradish peroxidase-conjugated anti-rabbit antibody (1:5000; Cat # NA934, GE Healthcare, Chicago, IL, USA) or horseradish peroxidase-conjugated anti-mouse antibody (1:20,000; Cat # NA931, GE Healthcare) was applied as the secondary antibody for immunostaining and incubated for 2 h at room temperature. After washing with PBS-T, a chemiluminescent substrate (Cat # WBKLS0500, Merck Millipore) was applied to detect the bands. The images were analyzed with ImageJ (NIH). The experiments were repeated 4 times.

Western blotting was performed for exosome samples to examine whether an exosome-characteristic marker protein (CD63) was observed. The exosome pellets were lysed with RIPA buffer (188-02451; Fujifilm Wako Pure Chemical, Osaka, Japan) containing a protease inhibitor cocktail (11697498001; Merck-Roche) and quantified using a BCA protein assay kit (23225; Thermo Fisher Scientific). A 5-µg sample of denatured proteins was subjected to SDS-PAGE and transferred onto a PVDF membrane (IPVH07850; Merck Millipore). The membrane was blocked with TBS buffer containing 0.05% Tween 20 and 3% BSA for 1 h at room temperature and incubated overnight at 4 °C with anti-CD63 primary antibody (1:2500; ab134045; abcam, Cambridge, UK). After incubation with an HRP-conjugated secondary antibody (1:2000; 7074; Cell Signaling Technology), the chemiluminescence signal was detected using a Novex ECL Chemiluminescent Substrate Regent kit (WP20005; Thermo Fisher Scientific), and the images were captured with LAS-3000 (Fujifilm, Tokyo, Japan). A change in the amount of CD63 is not thought to imply a change in the amount of exosomes. In the present study, the amount of exosomes used in the present experiments was defined as the total protein content. We noted that although CD63 is an abundant protein found in exosomes, it cannot be used as an intrinsic reference. The Minimal Information for Studies of Extracellular Vesicles 2018 (MISEV2018) specified that there is no universal exosome marker [9]. There are some reports showing quantitative changes in CD63 in exosomes obtained from the same cells with different siRNA transfection [10].

Phosphorylation of AKT protein, which is involved in the angiogenesis process, in HUVEC cells was confirmed by Western blotting [11,12,13]. HUEhT-1 cells, after the treatment of the exosomes for 48 h, were washed with PBS and lysed with RIPA buffer containing a protease inhibitor cocktail and a phosphatase inhibitor cocktail (Nacalai Tesque). Protein extracts were collected and quantified using a BCA protein assay kit. A 5-µg sample of denatured proteins was subjected to SDS-PAGE and transferred onto a PVDF membrane. The membrane was blocked with TBS buffer containing 0.05% Tween 20 and 3% BSA for 1 h at room temperature and incubated overnight at 4 °C with anti-phospho-AKT (Ser473) primary antibody (1:1000; 4060; Cell Signaling Technology) and anti-GAPDH antibody (1:3000; 010-25521; Fujifilm Wako Pure Chemical). After incubation with an HRP-conjugated secondary antibody (1:2000; 7074; Cell Signaling Technology for p-AKT and 1:1500; NA931; Amersham Biosciences (Amersham, UK) for GAPDH), the chemiluminescence signal was detected using a Novex ECL Chemiluminescent Substrate Regent kit (WP20005; Thermo Fisher Scientific). After observation of p-AKT, the membrane was incubated in a stripping buffer (2.0 M glycine buffer (pH 2.2) containing 0.1% SDS and 1% Tween20) and then incubated with anti-AKT (pan) primary antibody (1:1000; C67E7; Cell Signaling Technology). After incubation with an HRP-conjugated secondary antibody, the chemiluminescence signal was detected using a Novex ECL Chemiluminescent Substrate Regent kit. Images were analyzed with LAS-3000 (Fujifilm), and the relative ratio was calculated in comparison to the GAPDH. The experiments were repeated 5 times.

### 2.4. Ultrasensitive Thio-NAD Cycling ELISA

To detect trace amounts of GRP78 in the exosomes, an ultrasensitive thio-NAD cycling ELISA was performed [14,15]. This ELISA is coupled with a thio-NAD cycling reaction, and thus, the signal obtained from a sandwich ELISA using a primary and a secondary antibody is amplified by the thio-NAD cycling reaction, as previously described [16,17]. Briefly, we used a GRP78 ELISA kit (sandwich ELISA; Human HSPA5/GRP78/BiP ELISA Kit; LS-F11578, LifeSpan Biosciences, Seattle, WA, USA) and thio-NAD cycling. Although the kit includes streptavidin-HRP for the detection of GRP78, we used streptavidin-alkaline phosphatase for the thio-NAD cycling reaction at the detection step in our ultrasensitive measurement. The GRP78 standards contained in the kit and the exosome samples were incubated in primary GRP78 antibody-bound 96-well plates for 2 h at 37 °C. For comparison of GRP78 concentrations in the exosomes, the total protein concentrations in the exosomes collected from GRP78-OE, GRP78-KD, and GRP78-mock AGS cells were adjusted to 5 μg total protein/100 μL by BCA kit (Cat # 23227, Thermo Fisher Scientific). After the solution was aspirated, the secondary antibody (anti-GRP78 biotinylated antibody) of the ELISA kit was added, and the solution was incubated for 1 h at 37 °C. The microplate was washed with a wash buffer (TBS buffer containing 0.05% Tween 20) before adding streptavidin-alkaline phosphatase (41075-8; Mabtech, Nacka Strand, Sweden) diluted to 1:1000 in TBS buffer containing 0.1% BSA and 0.02% Tween 20, and then shaken for 1 h at room temperature. After the solution was removed by aspiration, 100 μL of thio-NAD cycling solution was added to each well. This solution contained 1.0 mM NADH (10107735001; Roche, Basel, Switzerland), 3.0 mM thio-NAD (44104001; Oriental East, Tokyo, Japan), 0.1 mM 17β-methoxy-5β-androstan-3α-ol 3-phosphate (provided by one of the authors [T.Y.]), and 30 U/mL 3α-hydroxysteroid dehydrogenase (T-58; Asahi Kasei Pharma, Tokyo, Japan) in 100 mM Tris-HCl (pH 9.5) [18]. Absorbance at 405 nm for the accumulated thio-NADH was measured using a microplate reader SH-1000 (Corona Electric, Ibaraki, Japan) every 5 min for 1 h at 37 °C, and the 405 nm signals were normalized to those at 660 nm [19]. For the linear calibration curves, the experimental data were obtained by subtracting the mean value of the blank signals from each of the corresponding measured datapoints.

### 2.5. Tube Formation Assay

The HUEhT-1 cells were incubated with 25 µg/mL exosomes for 48 h. After the exosome treatment, the HUEhT-1 cells were harvested in media using exosome-depleted FBS (Invitrogen). The tube formation assay was performed using an Endothelial Tube Formation Assay (In Vitro Angiogenesis) kit (CBA-200; Cell Biolabs, San Diego, CA, USA). HUEhT-1 cells were seeded at 2.5 × 10^5^ cells in T-25 culture flasks with 3 mL medium and incubated overnight at 37 °C. The medium was changed to exosome-containing medium. After 48 h, HUEhT-1 cells were seeded onto Matrigel in the Endothelial Tube Formation Assay kit using 96-well plates at 2.0 × 10^4^ cells/well and incubated overnight at 37 °C. Tube formation was examined by phase-contrast microscopy (IX71, Olympus, Tokyo, Japan), and tubes were analyzed with the Angiogenesis Analyzer plug-in on ImageJ. The data are representative of 12 independent experiments.

### 2.6. MTT Assay

The HUEhT-1 cells were seeded at 1 × 10^4^ cells/well in 96-well culture plates with 100 µL medium and incubated overnight at 37 °C. Exosome samples were applied to the cells. After 48 h, 10 µL of an MTT labeling reagent in an MTT Cell Count Kit (Cat #23506, Nacalai Tesque) was added to each well, and the cells were incubated for 4 h at 37 °C. The 100-µL solubilization buffer in the kit was then added to each well, and the plates were incubated overnight at 37 °C. Absorbance was measured at 570 nm and 405 nm, as its reference wavelength, with a microplate reader (SH1000, Corona Electric).

### 2.7. Wound Healing Assay

The HUEhT-1 cells were seeded at 2.5 × 10^5^ cells in T-25 culture flasks with 3 mL medium and incubated overnight at 37 °C. The medium was changed to exosome-containing medium. After 48 h, the HUEhT-1 cells were seeded into 2-well cell culture inserts (ibidi, Gräfelfing, Germany) on plates at 3.15 × 10^4^ cells/well and incubated overnight at 37 °C. The culture cell inserts were removed from the plate, and the fresh exosome-free medium was treated. The cells were examined with a phase-contrast microscope (CKX53, Olympus) and analyzed with the wound-healing size tool plugin on ImageJ [20]. The data are representative of nine independent experiments. 

### 2.8. Statistical Analysis

The data are expressed as mean ± SD. Significant differences were defined as *p* < 0.05 using 1-way ANOVA, followed by a post hoc Holm test for the ELISA and MTT assay data and the Kruskal-Wallis test, followed by a post hoc Holm test for the others, using *R* version 4.2.1 (R Foundation for Statistical Computing, Vienna, Austria). We obtained the number of experiments (*n*) as a multiplication of the biological and technical replicates.

## 3. Results

### 3.1. Exosome Characterization and GRP78 Quantification in Exosomes

**Figure 1:**
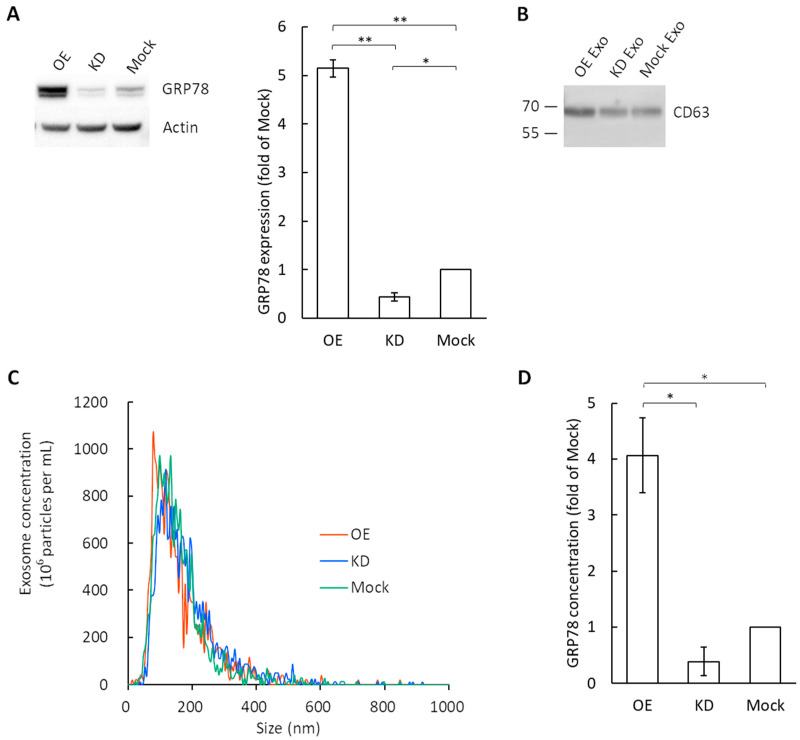
Characterization of exosomes isolated from the cell culture media of transfected gastric cancer AGS cells using a combination of a polymer precipitation method and an ultra-filtration method. (**A**) Western blotting of GRP78 in GRP78-OE, GRP78-KD, and GRP78-mock cells of AGS. Actin was used as the control. (**B**) Western blotting of an exosome-marker protein, CD63, for the exosomes collected from GRP78 overexpression (OE), knockdown (KD), and mock cells of AGS. (**C**) Exosome size distribution was determined by ZetaView. The peak values were around 124 nm in GRP78-OE cells, 118 nm in GRP78-KD cells, and 132 nm in GRP78-mock cells. (**D**) Comparison of concentrations of GRP78 in exosomes collected from GRP78-OE, -KD, and -mock cells of AGS. The data are expressed as a fold of the concentration of mock cells; * and ** indicate *p* < 0.05 and *p* < 0.01, respectively.

We confirmed that the commercially available, easy-to-use kit, which was mentioned in Section 2.2., was useful for exosome isolation [3,4]. Using this kit, the exosomes were collected from the culture medium of GRP78-OE, GRP78-KD, and GRP78-mock AGS cells. The expression levels of GRP78 in these 3 cell types were examined by Western blotting (Figure 1A). To confirm the presence of an exosome-specific marker, CD63, for the collected exosomes, Western blotting was performed for CD63 (Figure 1B). The bands around 70 kDa were observed for all 3 types of exosomes (i.e., OE, KD, and mock). The expression of CD63 is expected to show bands in the range of 26 kDa to 100 kDa because of the glycosylation of CD63 [21,22,23,24].

A nanoparticle tracking analysis with ZetaView revealed that the peak value of vesicles collected from the GRP78-OE AGS cells was 124 nm, that of GRP78-KD AGS cells was 118 nm, and that of GRP78-mock AGS cells was 132 nm (Figure 1C). These two lines of evidence confirmed that the vesicles we obtained were exosomes and were homogeneous. The numbers of particles per µg of total protein were calculated to be 2.06 × 10^8^ particles/µg for GRP78-OE, 2.94 × 10^8^ particles/µg for GRP78-KD, and 2.01 × 10^8^ particles/µg for GRP78-mock. Therefore, it is reasonable to use the total protein content as the reference for normalization in subsequent experiments.

As shown previously, the limit of detection (LOD) of our ultrasensitive thio-NAD cycling ELISA for GRP78 was calculated from calibration curves using standard GRP78 protein samples, and it was 1.44 × 10^−19^ moles/assay using a 100-μL volume for one assay [3]. The typical GRP78 concentration in the exosomes collected from GRP78-OE, -KD, and -mock AGS cells was 3.75 pg/mL, 0.29 pg/mL, and 1.06 pg/mL, respectively. Here, the total protein masses in the exosomes were measured using the BCA method and adjusted for comparison of the GRP78 concentrations. The GRP78 content was greater in the exosomes derived from GRP78-OE AGS cells than in those derived from GRP78-KD and GRP78-mock AGS cells (Figure 1D).

### 3.2. Tube Formation Assay of Vascular Endothelial Cells by the Application of GRP78-Containing Exosomes

**Figure 2:**
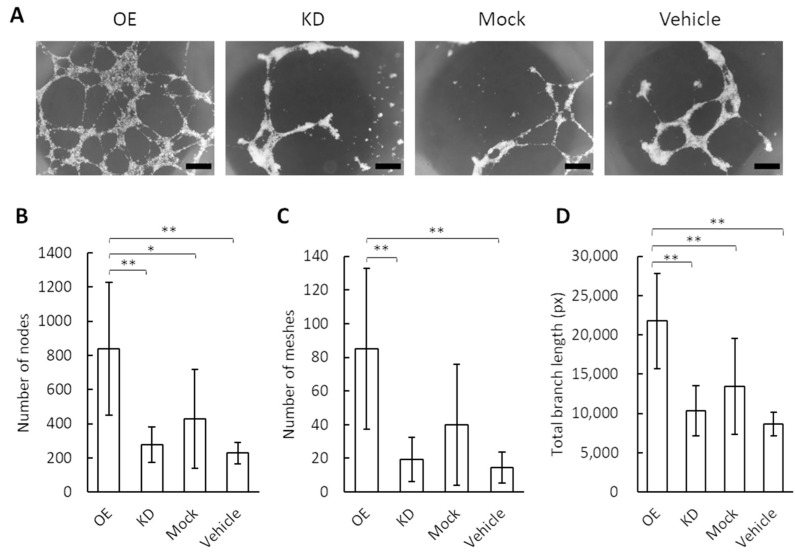
Tube formation assay for vascular endothelial cells by the application of exosomes containing various concentrations of GRP78. (**A**) Photos of tube formation. OE, KD, mock, and vehicle indicate the application of exosomes collected from GRP78-overexpression AGS cells, those from GRP78-knockdown cells, those from GRP78-mock cells, and the application of culture medium, respectively. Scale bar = 500 µm. (**B**) The number of nodes in an area of 9.27 mm^2^; *n* = 12 each. (**C**) The number of meshes in an area of 9.27 mm^2^; *n* = 12 each. (**D**) Total branch length (pixels analyzed with ImageJ) in an area of 9.27 mm^2^; *n* = 12 each; * and ** indicate *p* < 0.05 and *p* < 0.01, respectively.

Endothelial cells are induced to differentiate and form tube-like structures when activated by a angiogenic signal [25]. To obtain direct evidence that the higher concentration of GRP78 contained in the exosomes can affect angiogenesis, we performed a tube formation assay in the vascular endothelial cells using the exosomes derived from GRP78-OE, -KD, and -mock AGS cells (Figure 2A). The number of nodes in the endothelial cells was greater following the application of the GRP78-OE exosomes compared with the application of the GRP78-KD and GRP78-mock AGS cells (Figure 2B). In addition, the number of meshes and the branch length in the endothelial cells were greater and longer, respectively, following the application of GRP78-OE exosomes compared with GRP78-KD and GRP78-mock AGS cells (Figure 2C,D). These results showed that endothelial cell tubes are easily produced by applying high concentrations of GRP78-containing exosomes.

### 3.3. Phosphorylation of AKT in Vascular Endothelial Cells by the Application of Grp78 Containing Exosomes

**Figure 3:**
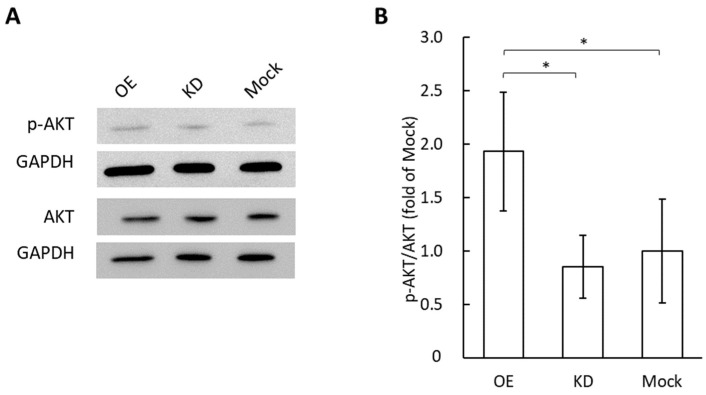
Phosphorylation of AKT in vascular endothelial cells after the treatment of exosomes containing various concentrations of GRP78 for 48 h. (**A**) Western blotting for phosphorylated and non-phosphorylated AKT. GAPDH was used as the control. (**B**) Comparison of the Ser473 phosphorylation state ratio of AKT estimated from the band density; *n* = 5 each; * *p* < 0.05.

Tube formation in vascular endothelial cells occurs in an AKT-dependent manner, and many previous studies demonstrated that AKT phosphorylation, but not other angiogenic mediators, is required for angiogenesis [26,27,28]. Therefore, we performed Western blotting experiments for phosphorylated AKT in vascular endothelial cells after applying exosomes derived from GRP78-OE, -KD, and -mock AGS cells (Figure 3). The phosphorylation of AKT was significantly greater with the application of GRP78-OE exosomes compared with GRP78-KD and GRP78-mock AGS cells.

### 3.4. MTT Assay for Vascular Endothelial Cells by the Application of GRP78-Containing Exosomes

**Figure 4:**
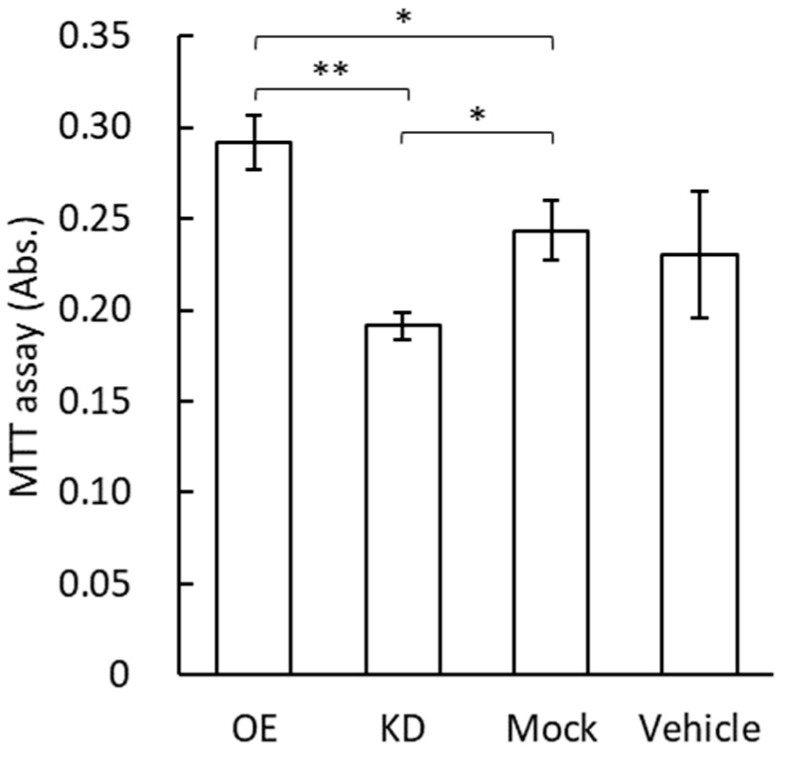
The MTT assay for vascular endothelial cells by the application of exosomes containing various concentrations of GRP78. The *y*-axis indicates absorbance of 570 nm, showing that the higher the absorbance, the more viable the cells; *n* = 4 each; * and ** indicate *p* < 0.05 and *p* < 0.01, respectively.

The cell proliferation rate of vascular endothelial cells after the application of exosomes containing GRP78 was examined using an MTT assay (Figure 4). The high concentration of GRP78 contained in the exosomes of the GRP78-OE AGS cells was expected to increase endothelial cell proliferation. The MTT assay showed that the number of viable endothelial cells was significantly increased by the application of GRP78-OE exosomes compared with GRP78-KD and GRP78-mock exosomes (Figure 4). There is also a significant difference between GRP78-KD and GRP78-mock exosomes (Figure 4).

### 3.5. Wound Healing Assay for Migration Potential of Vascular Endothelial Cells Following the Application of GRP78-Containing Exosomes

**Figure 5:**
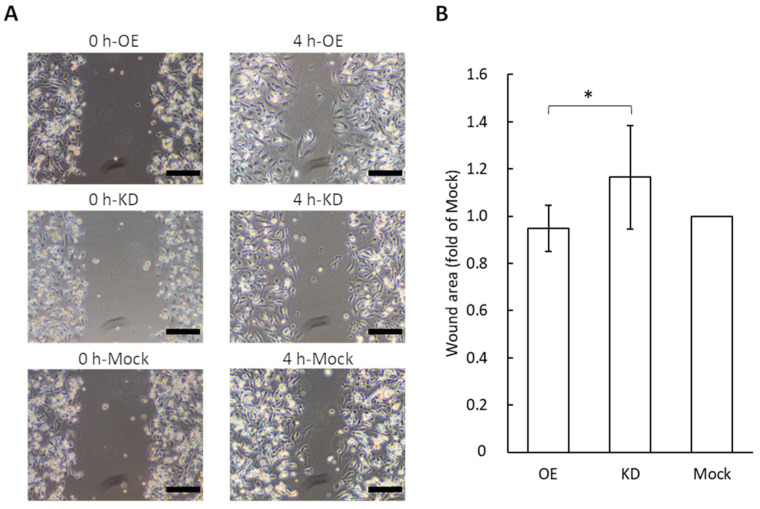
Wound healing assay for vascular endothelial cells by the application of exosomes containing different concentrations of GRP78. (**A**) Typical photos 4 h after wound healing assays for HUEhT cells after application of exosomes; scale bar = 250 μm. (**B**) Comparison of wound area. The wound area is small, indicating that the cell motility is high; *n* = 9 each; * *p* < 0.05.

A change in the cell migration potential for vascular endothelial cells was examined after the application of different concentrations of GRP78 in exosomes derived from AGS cells (Figure 5). We used a wound healing assay that shows how cell-free areas are filled by the migration of cells. The application of exosomes collected from GRP78-OE AGS cells diminished the wound area compared to wounds treated with GRP78-KD exosomes. That is, the cell migration capacity of endothelial cells increased with the application of the GRP78-OE exosomes. This result is consistent with those of a previous study showing that the artificial activation of AKT in endothelial cells promotes cell migration [26]. The results indicated that exosomal GRP78 promoted endothelial cell motility.

## 4. Discussion

In the present study, we prepared GRP78-OE and GRP78-KD AGS cells, which are gastric cancer cells. We successfully quantified the concentration of GRP78 in GRP78-rich exosomes collected from GRP78-OE AGS cells using our ultrasensitive thio-NAD cycling ELISA, and then incubated these exosomes with vascular endothelial cells. Previous studies revealed that GRP78 on the cell surface is involved in AKT phosphorylation [29]. Because we hypothesized that the angiogenic effect occurs via AKT proteins, we used an ELISA assay that can measure GRP78 on exosome membranes. Our results indicated that tube formation was more advanced when the GRP78-OE exosomes were applied, compared with GRP78-KD and GRP78-mock exosomes. The Ser473 phosphorylation state ratio of AKT, which is involved in angiogenesis, was increased by applying the GRP78-OE exosomes to endothelial cells. The MTT assay showed that GRP78-OE exosome treatment increased the proliferation rate of endothelial cells, and the wound healing assay showed that this treatment increased the migration capacity of the endothelial cells. Together, the results clearly demonstrated that GRP78-rich exosomes promote the angiogenesis of vascular endothelial cells.

There were no significant differences between KD and mock in some experiments. This issue occurred because the amount of GRP78 in the exosomes did not differ much between KD and mock (Figure 1D). To support this fact, the amount of GRP78 was hardly different between GRP78-KD and GRP78-mock AGS cells, as shown by Western blotting of the cell lysates (Figure 1A). This can be also seen in previous studies using other cells. It was demonstrated that the GRP78 suppression ratio by shRNA (i.e., KD) in pancreatic cancer cells was clearly smaller than the GRP78 overexpression ratio [30]. Furthermore, the GRP78 suppression ratio was also smaller than the GRP78 overexpression ratio in colon cancer cells [31]. The reason for this small KD ratio is due to the GRP78 gene being essential for survival. Homozygous GRP78 null genotype mice are known to die during development [32]. In other words, it is considered that an expression of GRP78 easily increases under the unfolded protein response environment, even though the knockdown is processed in cancer cells. There are some previous studies comparing GRP78 OE and mock. For example, Wang et al. showed a change in angiogenic function by overexpressing GRP78 in vascular endothelial cells [33], and Kim et al. showed that transplantation therapy of human mesenchymal stem cells overexpressing GRP78 promoted angiogenesis through intercellular communication [34]. For all these reasons, the data obtained in the present study regarding the comparisons among KD, OE, and mock are very reasonable and contain sufficient physiological significance. This is based on the idea that quantitative rather than qualitative changes in the amount of GRP78 are important, and thus we performed quantification using an ultrasensitive ELISA method.

The GRP78-KD cells used in the present study were produced by Taiwan Academia Sinica, and the most effectively knock-downed cell line was selected when the shRNA gene transfection was performed. The cells in which the same shRNA was applied were used in the previous studies for mouse xenografts [2]. Thus, knockdown was thought to be successful in the GRP78-KD AGS cells. However, we have not completely ruled out the off-target protein knockdown. Therefore, we will continue to pursue our research with additional caution. The controlled shRNA (referred to as mock in our study) is the plasmid that cannot generate shRNA to target GRP78. The knockdown efficiency was revealed in our present data (Figure 1D). Our results showing that the typical GRP78 concentration in exosomes collected from GRP78-OE, -KD, and -mock AGS cells were 3.75 pg/mL, 0.29 pg/mL, and 1.06 pg/mL, respectively, indicated that the expression of exosomal GRP78 in KD-AGS cells was much lower than in the mock cells. Although GRP78 is an important heat shock protein to facilitate cancer cell adaptation to the stress environment [1], the exosome experiments with no significant differences between GRP78-KD and mock groups in AKT phosphorylation suggest that some other proteins may be involved in the activation of the AKT signaling pathway, upregulating p-AKT in the GRP78-KD cells. The detailed mechanisms need to be further investigated.

The role of AKT phosphorylation in the angiogenic process must be considered. In the presence of vascular endothelial growth factor (VEGF), the PI3K-AKT pathway plays an important role in angiogenesis [27,35,36,37]. On the other hand, in previous studies in which the AKT phosphorylation site was mutated, angiogenesis did not occur, even if VEGF was present, whereas angiogenesis did occur in other studies that evoked AKT activation, even if VEGF was absent [26,28,38]. The addition of angiogenic mediators (e.g., Gas6: a protein in exosomes secreted from perivascular cells) to vascular endothelial cells increases AKT phosphorylation [13]. On the other hand, the application of angiogenesis inhibitors to vascular endothelial cells decreases AKT phosphorylation [7]. Angiogenesis is suppressed when AKT inhibitors are included in the co-culture model of lung cancer cells and HUVEC [12]. The stress fiber formation with cytoskeletal actin is suppressed in vascular endothelial cells in which the AKT phosphorylation site is inhibited, whereas it is enhanced in endothelial cells in which AKT is forcibly phosphorylated; these results are the same, regardless of the presence or absence of VEGF [28].

Here, the relationship between GRP78 and AKT phosphorylation is considered. Previous studies revealed that GRP78 on the cell surface is involved in AKT phosphorylation [29]. In endothelial cells, GRP78 was reported to be related to AKT-dependent angiogenesis, but not the VEGF-dependent mechanism [39]. Endothelial cell migration is due to AKT phosphorylation [40]. Regarding the wound healing assay in our study, GRP78-OE exosomes promoted cell migration compared with GRP78-KD exoisomes, indicating that exosomal GRP78 plays a role in endothelial cell migration. Furthermore, the inhibition of GRP78 on the cell surface by its antibodies prevents the function of AKT and its subsequent cascade [41]. In prostate cancer, GRP78-KD mice exhibit the suppression of phosphatase and tensin homolog (PTEN)-mediated activation of AKT (i.e., through the PI3K-AKT pathway) [42]. Furthermore, GRP78 colocalizes with active AKT [43]. In previous studies, the naked GRP78 causes Akt phosphorylation, and thus, angiogenesis via exosomes is thought to occur in the same pathway [29]. However, we would like to claim that considering the possibility of proteolysis as well as the distance and efficiency of the horizontal propagation of information, it is important to consider GRP78 propagation via exosomes.

In the future, it will be necessary to investigate whether angiogenesis occurs by the application of GRP78-rich exosomes in vivo. In addition, we expect that GRP78 on the exosome surface is transferred to the surface of vascular endothelial cells and acts on AKT, resulting in angiogenesis; however, where GRP78 is transported to and the reaction pathway underlying the GRP78 activation of AKT remains unknown. To address these issues, our recent work to measure GRP78 separately in the membrane and lumen of the exosomes may be useful [3]. Moreover, we have no direct knowledge of whether GRP78-induced angiogenesis is independent of VGEFs. Determining whether exosomal GRP78 stimulates the secretion of VEGF in vascular endothelial cells, acting in an autocrine/paracrine manner to cause angiogenesis, should be further investigated.

## 5. Conclusions

We successfully quantified proteins in exosomes using a thio-NAD cycling ELISA. Until now, the quantification of exosomal proteins has been difficult. To the best of our knowledge, this is the first study providing quantitative data that exosomal GRP78 promotes angiogenesis. This study provides new insights into the construction of the tumor microenvironment through cell-cell communication by the exosomes.

## Data Availability

All data that support the findings of this study are available from the corresponding authors upon reasonable request.
